# Supplementary material for: MetaRibo-Seq measures translation in microbiomes
Source: Nat Commun. 2020 Jun 29;11:3268. doi: 10.1038/s41467-020-17081-z (PMC7324362; doi:10.1038/s41467-020-17081-z)
Supplement: Supplementary file 10 — Supplementary Data 7 [file 41467_2020_17081_MOESM10_ESM.zip › File2/Confidence_High_Taxonomy/156129_out.krona.html]

Javascript must be enabled to view this page.

members
magnitude
magnitudeUnassigned
count
unassigned
taxon
rank

156129\_out

147

2
147
superkingdom

147
phylum
976

200643
class
147

171549
147
order

family
147
815

816

SRS016381\_contig\_number\_contig-100\_345.29950SRS049896\_contig\_number\_22010SRS052027\_contig\_number\_14722SRS075773\_contig\_number\_22396
147
genus
4


SRS065176\_contig\_number\_9018
162156
1
species


SRS063928\_contig\_number\_1270
821
1
species

2292280

SRS147022\_contig\_number\_696
1
species


SRS074670\_contig\_number\_6462SRS143780\_contig\_number\_3059
818
species
2

species
10
556259

SRS012902\_contig\_number\_1383SRS023829\_contig\_number\_679SRS045826\_contig\_number\_21683SRS065504\_contig\_number\_22366SRS075398\_contig\_number\_3203SRS077502\_contig\_number\_contig-100\_1173.104769SRS077849\_contig\_number\_22086SRS1041037\_contig\_number\_contig-100\_1271.89947SRS143876\_contig\_number\_21513SRS144362\_contig\_number\_11846

2292956

SRS018133\_contig\_number\_2442SRS018427\_contig\_number\_30704SRS893383\_contig\_number\_contig-100\_468.198617
species
3

371601

SRS013800\_contig\_number\_25340SRS015217\_contig\_number\_21687SRS020622\_contig\_number\_962SRS148091\_contig\_number\_4576
species
4

29
species

SRS012849\_contig\_number\_27886SRS013951\_contig\_number\_27177SRS014923\_contig\_number\_35326SRS015694\_contig\_number\_23272SRS016056\_contig\_number\_7811SRS016267\_contig\_number\_10362SRS016335\_contig\_number\_6916SRS016954\_contig\_number\_19212SRS019161\_contig\_number\_1810SRS019787\_contig\_number\_3788SRS023346\_contig\_number\_1655SRS045713\_contig\_number\_2598SRS045739\_contig\_number\_2449SRS050925\_contig\_number\_16202SRS051882\_contig\_number\_10262SRS057717\_contig\_number\_168SRS058268\_contig\_number\_162SRS062654\_contig\_number\_20751SRS063190\_contig\_number\_12715SRS077127\_contig\_number\_10542SRS1041134\_contig\_number\_3570SRS1055022\_contig\_number\_11804SRS1055049\_contig\_number\_13830SRS1055056\_contig\_number\_contig-100\_875.73073SRS140645\_contig\_number\_1703SRS143991\_contig\_number\_16815SRS149244\_contig\_number\_4459SRS893259\_contig\_number\_contig-100\_1090.54921SRS893288\_contig\_number\_1987
469590

1
species
291645

SRS1041142\_contig\_number\_66

species
1

SRS016753\_contig\_number\_669
291644

1
species
1263050

SRS014412\_contig\_number\_6558

9
species
457390

SRS046068\_contig\_number\_contig-100\_1718.36464SRS056537\_contig\_number\_contig-100\_1001.326627SRS062701\_contig\_number\_7030SRS1055038\_contig\_number\_485SRS143085\_contig\_number\_contig-100\_1215.47480SRS147977\_contig\_number\_contig-100\_1357.85002SRS148784\_contig\_number\_contig-100\_1363.162202SRS148970\_contig\_number\_contig-100\_1430.37216SRS893292\_contig\_number\_contig-100\_1275.62976

1
species
46506

SRS097920\_contig\_number\_26749

47678

SRS014287\_contig\_number\_18007SRS049773\_contig\_number\_125
species
2

1
species
820

SRS893279\_contig\_number\_1672

species
4

SRS012273\_contig\_number\_21319SRS017859\_contig\_number\_1170SRS022071\_contig\_number\_32793SRS148721\_contig\_number\_54640
1946086

28116

SRS013215\_contig\_number\_2266SRS013382\_contig\_number\_1940SRS013476\_contig\_number\_30386SRS013638\_contig\_number\_10419SRS014736\_contig\_number\_8331SRS015578\_contig\_number\_2394SRS015782\_contig\_number\_24625SRS015794\_contig\_number\_3254SRS015890\_contig\_number\_6935SRS016132\_contig\_number\_3590SRS017622\_contig\_number\_121SRS017701\_contig\_number\_10710SRS017821\_contig\_number\_20154SRS018623\_contig\_number\_65SRS018817\_contig\_number\_15042SRS018936\_contig\_number\_9920SRS018984\_contig\_number\_1679SRS019178\_contig\_number\_1065SRS019397\_contig\_number\_30SRS020271\_contig\_number\_3875SRS020328\_contig\_number\_35062SRS020869\_contig\_number\_5154SRS023526\_contig\_number\_14757SRS024132\_contig\_number\_21622SRS024388\_contig\_number\_1559SRS044535\_contig\_number\_10925SRS045244\_contig\_number\_35SRS050422\_contig\_number\_14837SRS050752\_contig\_number\_12470SRS052576\_contig\_number\_contig-100\_702.16211SRS053649\_contig\_number\_11411SRS057480\_contig\_number\_8357SRS058070\_contig\_number\_7137SRS058770\_contig\_number\_16152SRS062427\_contig\_number\_9895SRS063518\_contig\_number\_14673SRS064232\_contig\_number\_3871SRS075341\_contig\_number\_contig-100\_835.154615SRS075984\_contig\_number\_27304SRS077454\_contig\_number\_13995SRS078419\_contig\_number\_8128SRS097889\_contig\_number\_2566SRS098655\_contig\_number\_9216SRS098881\_contig\_number\_13908SRS1041164\_contig\_number\_2448SRS104327\_contig\_number\_4907SRS1054928\_contig\_number\_3332SRS1055076\_contig\_number\_9885SRS1055083\_contig\_number\_8331SRS1055095\_contig\_number\_1517SRS142542\_contig\_number\_20134SRS142980\_contig\_number\_contig-100\_1510.47329SRS143148\_contig\_number\_15161SRS143466\_contig\_number\_18050SRS143523\_contig\_number\_contig-100\_1728.139479SRS143722\_contig\_number\_716SRS145308\_contig\_number\_3027SRS145497\_contig\_number\_16925SRS146764\_contig\_number\_30454SRS146812\_contig\_number\_37738SRS147039\_contig\_number\_4186SRS147614\_contig\_number\_3925SRS147653\_contig\_number\_6271SRS148817\_contig\_number\_10067SRS148874\_contig\_number\_13983SRS150029\_contig\_number\_550SRS893231\_contig\_number\_3087SRS893285\_contig\_number\_715SRS893341\_contig\_number\_5995SRS893342\_contig\_number\_11167SRS893369\_contig\_number\_1038SRS971276\_contig\_number\_23831
species
72
